# The relationship between personality dimensions, spirituality, coping strategies and clinical clerkship satisfaction among intern nursing students: a cross-sectional study

**DOI:** 10.1186/s12912-020-00469-z

**Published:** 2020-08-07

**Authors:** Yasser Rezapour-Mirsaleh, Mahdi Aghabagheri

**Affiliations:** 1 Department of Counseling, Faculty of Humanities & Social Sciences, Ardakan University, P.O. Box 184, Ayatollah Khatami Blvd., Ardakan, Yazd, Iran; 2 Medical Education Evaluation Center, Health Ministry, Eskandari St, Tehran, Iran

**Keywords:** Clinical clerkship, Intern nursing students, Personality, Coping, Spirituality

## Abstract

**Background:**

The clinical clerkship is a crucial stage in nursing training, yet a dissatisfaction at this stage may cause a nurse to quit in future. This study aimed to investigate the relationship among personality dimensions, spirituality, coping strategies and clinical clerkship satisfaction among intern nursing students.

**Methods:**

This was a correlational, cross-sectional study. A total of 293 Iranian nursing students, who were fulfilling their clinical clerkship at university-affiliated hospitals in Tehran, were selected using cluster random sampling. All participants were provided with standard questionnaires including personality dimensions (NEO_FFI), spirituality, coping strategies (WoCQ), and satisfaction with clinical clerkship. Data were analyzed using Pearson correlation and hierarchical multiple regression.

**Results:**

The results showed that spirituality (β = 0.32, *p* < 0.001), problem-focused coping (β = 0.26, *p* < 0.001) and extraversion (of personality dimensions, β = 0.22, *p* < 0.001) played significant roles in predicting clinical clerkship satisfaction among intern nursing students. Regression analysis showed openness (β = 0.14, *p* < 0.01), extraversion (β = 0.16, *p* < 0.01), and spirituality (β = 0.23, *p* < 0.001) could significantly predict problem-focused coping style among intern nursing students. However, personality dimensions and spirituality were not good predictors for emotion-focused coping among these students (*p* > 0.05).

**Conclusion:**

Personality dimensions, coping strategies and, in particular, spirituality are good predictors of clinical clerkship satisfaction among intern nursing students. Therefore, paying attention to spiritual needs in nursing students, improving their problem solving skills in dealing with clinical stress and training extraversion characteristics can be effective in enhancing their satisfaction with clinical clerkship.

## Background

Clinical clerkship is an essential part of nursing education providing the students with opportunities to work with real patients. Clinical experiences play a key role in forming professional identity of nursing students, and the quality of clinical clerkship is a significant factor in determining the efficacy of nursing education [1]. The gap between theory and practice is one of the major problems for nursing students during clinical clerkship [2]. This phenomenon is more evident among the students who have just entered the clinical setting. In particular, problems arise when there is a transition from the academic environment into the clinical practice setting where nursing students deal with the so-called “reality shock” [3].

Reality shock is defined as a contradiction that nurses encounter when they enter the clinical setting; this contradiction is raised when intern nursing students are faced with a mismatch between the principles or ideals they have acquired at the university and the situations that they experience in reality in clinical setting. This might cause a feeling of incompetency in students while caring real patients and reduces their satisfaction with the clinical clerkship [4].

### Satisfaction with clinical clerkship

Nursing education programs in Iran consist of a 4-year undergraduate education and then, students are certified with bachelor degrees. The senior students exclusively devote themselves to clinical clerkship delivered in hospitals affiliated to universities. In doing so, students are provided with opportunities to deal with health-care environment in most wards including intensive care and specialist wards. Students are under the direct guidance of nursing supervisors for the first 3 years of education followed by acquiring clinical skills in the last year [5].

In nursing education, a clerkship refers to the practice of clinical courses by nursing students during their final year of study. The prospective satisfaction with clinical clerkship plays an important role in deciding whether to stay in the nursing profession [6]. Therefore, to prevent nursing students from quitting and to help them grow more interested in nursing, investigating clinical clerkship satisfaction in nursing students who have just entered clinical setting seems necessary [7].

However, the evidence suggests that if there are underlying factors, satisfaction with the clinical clerkship can encourage nursing students to remain in the nursing profession [8]. Thus, investigating the factors impressing clinical clerkship satisfaction in the nursing students, during a period they have just entered the clinical setting, can be beneficial to their proper transition from this stage.

### Personality dimensions

Evidence shows there is a relationship between personality and interest in nursing [9]. Personality characteristics are predictors of burnout and program completion among intern nursing students [10]. Furthermore, previous studies have showed clinical performance of students is associated with their personality characteristics [11, 12]. Herein, to investigate personality dimensions of student nurses, Costa and McCrae’s “Big-Five-factor” theory of personality was employed. In “Five-Factor” model, personality dimensions are divided into five distinct categories as follows: neuroticism, extraversion, openness, agreeableness, and conscientiousness [13]. Neuroticism is defined as emotional instability and tendency to experience negative emotions, such as anger, anxiety, or depression. Extraversion is defined as the desire to have high social relationships, to communicate with others, and to express thoughts and feelings. Openness refers to the desire to gain new and unfamiliar experiences. These people are aware of their feelings and thoughts and are curious about new art, beauty, and emotions. Agreement means getting along with others and their different attitudes, being in harmony with the community, accepting others and being kind to people. Finally, conscientiousness refers to striving for success, accepting responsibility for things, controlling impulses, and planning in life [13]. Evidence shows neuroticism positively and extraversion, openness, agreeableness, and conscientiousness negatively correlate with nurses’ burnout [14] and compassion fatigue [15].

### Spirituality

Spirituality is a personal attempt to find meaning and purpose of life, to answer questions about existence, and to understand our relationship to the sacred or transcendent. Spirituality may (or may not) arise from religion and entails the abandonment of material values and the pursuit of transcendent values [16]. Although the role of spirituality and spiritual care in nursing has been explored, the role of spirituality in students’ satisfaction with clinical clerkships could be a significant area for speculation [17]. For those exposed to stressful situations like nurses, religion and spirituality seem beneficial so as to overcome the barriers. While a large body of evidence is available on spirituality and spiritual care of patients, a couple of studies have been done on the spirituality of nurses and their spiritual needs [18, 19]. Nurses’ understanding of spirituality is not confined to religion and their sense of spirituality is felt beyond being religious [20]. However, it can be stated that spirituality is such a multidimensional concept that various definitions have been put forward for it. Recent studies have revealed that spirituality is related to stress alleviation in nursing students [21] and nurses need to be provided with spiritual nourishment in their work environment [18]. Spirituality can lead or guide nurses to consider caring patients glorified as a sacred job and a way to meet their spiritual goals; therefore, spirituality can increase job satisfaction among nurses [22]. The findings of a study showed spirituality reduces the clinical practice stress in nursing students because it causes students to face stress with a transcendent attitude and find meaning in clinical practice [23].

### Coping strategies

Coping refers to attempt to manage one’s own thoughts and behaviors in stressful situations and reduce or tolerate stress and conflict. There are two general coping strategies: (a) problem-focused coping such as organized problem solving, connotating attempt to find the cause of a problem by gathering information, concentration, and decision making; and (b) emotion-focused coping such as escape-avoidance, connotating the regulation of aroused emotions to alleviate distress [24]. Although the general perception is that the problem-oriented coping style is merely associated with reduced stress, the consequential use of styles depends on the type of stressful situation. The emotion-focused coping can, for instance, be useful for stressors that seem uncontrollable, such as terminal illness diagnosis or grief [25, 26]. Upon entering clinical clerkship, students encounter many problems and stresses. These stresses can spring out of a gap between theory and practice, caring for dying patients, sensing uncertainty about clinical ability, inter-personal problems with patients, and overload of daily work [27, 28]. Recent studies have indicated effective coping with stress during clinical clerkship is largely associated with their clinical performance [29]. Although studies show students are more likely to use problem-oriented coping, especially as they approach the end of the clinical clerkship [8, 28], the use of both types of coping styles is seen during the clinical clerkship [29]. However, the emotion-focused coping style is more apparent when the nurse encounters situations that are out of control [8].

## Research framework

To succeed in the nursing profession, personality type is very important. Some people with certain personality traits perform better as a nurse [9]. For example, students who are extrovert have a better adaptation to nursing and higher clinical performance [8, 11]. Responsibility and openness are also associated with higher job performance and lower burnout [14]. In addition, personality dimensions can play a role in other characteristics that lead to better performance in nurses. Coping with clinical stresses is one of the skills associated with nurses’ personality [11, 30]. For example, extraversion and neuroticism play a part in coping strategies where nursing students typically deal with stressors [30]. However, apart from the nurses’ personality, because nursing is considered an altruistic profession, spirituality can also affect their professional performance [17]. Spirituality can also affect the way nurses cope with clinical stressors [21]. However, there is no evidence indicating spirituality plays a more prominent role in a person’s adaptation to nursing.

## Methods

A correctional descriptive research design using cross-sectional approach was employed to evaluate the relationship among personality dimensions, spirituality, coping strategies and clinical clerkship satisfaction among intern nursing students. On the other hand, this study is correlational in that it evaluates the relationship between variables and is a cross-sectional because analysis of the data occurs at a specific point in time. Firstly, clinical clerkship satisfaction was considered a criterion variable in which personality dimensions, coping strategies and spirituality were used as predictors. Secondly, coping strategies was regarded as a criterion variable where personality dimensions and spirituality were used as predictors. Therefore, the following hypotheses were proposed:

1. Personality dimensions, spirituality, and coping strategies can significantly predict clinical clerkship satisfaction among intern nursing students.
2. Personality dimensions and spirituality can significantly predict problem-focused coping strategy among intern nursing students.
3. Personality dimensions and spirituality can significantly predict emotion-focused coping strategy among intern nursing students.

### Participants and data collection

Research sample consisted of the undergraduate intern nursing students who were fulfilling their clinical clerkship at university-affiliated hospitals in Tehran, Iran. A cluster sampling method was employed; among all the universities admitting nursing students in Tehran, Iran, out of which five universities were randomly selected (Islamic Azad, Iran, Shahid Beheshti, Shahed, and Tehran University of Medical Sciences). Amongst intern nursing students in these universities, students were ascertained eligible if they were doing clinical practice at least 15 h a week. The total number of eligible students was 2100. The sample size, according to Krejcie and Morgan’s formula [31] was 324. Therefore, the number of students in each university was almost the same, i.e. 65 students per university. We prepared a list of eligible participants and using randomization software, 325 students were selected. Considering the university permission, the list of students was provided by the registration office. The list was numbered after which a random selection was made according to numbers. If a person did not want to participate and did not complete the consent form, he or she would be withdrawn and replaced by another student. Afterwards, researchers provided detailed explanations about research objectives, advantages, and how to fill out the questionnaires and consent form. The consent form was signed by all participants. In addition, they were reassured of the confidential terms of the information. Out of 325 distributed questionnaires, 32 were incomplete. The remaining 293 responses were valid and could be analyzed (90.1%). It seemed that the high number of incomplete questionnaires was due to the large number of items, which caused some of the items not to be or randomly answered. Along with the questionnaires, demographic information such as age, gender, hours of clerkship per week, residence status, and the use of university fund was collected. The questionnaires took approximately 25 min to be completed.

### Instruments

#### NEO five factor inventory (NEO-FFI-S)

The shortened form of NEO five factor inventory (NEO-FFI-S) is a 60-item questionnaire measuring five dimensions of the typical personality: Neuroticism (N), Extraversion (E), Openness (O), Agreeableness (A), and Conscientiousness (C) [32]. Respondents indicated their level of agreement to each item on a five-point Likert-type scale ranging from 1 (strongly disagree) to 5 (strongly agree). In this study, the authorized Persian translation of the NEO-FFI-S [33] was used. Validity and reliability of the Persian version of NEO-FFI-S was approved [33]. In the present study, Cronbach’s alpha for N, E,O, A and C were .92, .90, .86, .88 and .88, respectively.

#### Spirituality scale

Spirituality was measured utilizing a researcher-made questionnaire. This scale is composed of 15 items designed based on the definition of spirituality [16] and composed of ultimate questions about life, meaning of existence and relationship with the sacred or transcendent issues. Examples include: “I am more satisfied when helping the one in need of help although being in need of help myself” and “I have no doubt that the universe has a destination”. Score was assigned to each item based on a 5-point Likert-type scale ranging from 1 (strongly disagree) to 5 (strongly agree). Content and construct validity of the scale were confirmed by a pilot study. The Kaiser–Meyer–Olkin (KMO) was .84, Bartlett’s test of sphericity was X^2^ = 4316.36 (*p* < .001) and a total of 62.5% variance was explained by one-factor structure of the scale. There was a positive relationship between this scale and spirituality as well as spiritual care scale in nurses [34, 35]. In the present study, Cronbach’s alpha of this scale was 0.92.

#### Ways of coping questionnaire (WoCQ)

The Persian version of Folkman and Lazarus’ ways of coping questionnaire (WoCQ) [36] was used to measure coping styles of the participants. This is a 67-item instrument scored on a four-point Likert scale from ‘not used’ (1) to ‘used a great deal’ (4). The coping strategies were grouped into two styles of coping: problem-focused and emotion-focused coping styles. For example, “I made a plan of action and followed it” was an item of problem-focused style whereas “I tried to forget the whole thing” or “I slept more than usual” were instances of emotion-focused style. The problem-focused coping style including cognitive and behavioral efforts is based on problem solving and helps to change or control the stressful situation. On the other side, the emotion-focused coping style including cognitive and behavioral efforts helps to reduce or manage the stressful situation but it does not directly focus on problem solving. The Persian translation of the WoCQ has demonstrated good internal consistency as well as high test–retest reliability [37]. In the present study, Cronbach’s alpha for problem-focused style was 0.86 while it was 0.82 for the emotion-focused style.

#### Satisfaction with clinical clerkships questionnaire (SCCQ)

Satisfaction with clinical clerkships was measured utilizing a research-made questionnaire [38]. Items of this questionnaire were designed according to the Herzberg’s two factor theory of job satisfaction (motivational and hygienic factors) [39]. This is a 36-item instrument scored on a seven-point Likert scale ranging from 1 (strongly disagree to 5 (strongly agree). Some examples included: “The clinical clerkship made me confident in my decision to choose nursing as a job”, “I have a good clinical clerkship period” and “During my clinical clerkship, I enjoyed working with other students and my instructors”. The total score obtained from sum of the scores of all items showed a general satisfaction with clinical clerkships. Content and construct validity of this questionnaire were verified and Cronbach’s alpha coefficient was reported as 0.96 [38]. In the present study, Cronbach’s alpha of this questionnaire was 0.92.

### Data analysis

Statistical analyses were performed using SPSS version 16.0. Continuous variables were presented as mean ± standard error, and categorical variables as proportions. Participants’ responses to items of the same domain in the scale were used to replace missing values. The level of significance was considered to be 95% (*p* < .05). Pearson correlation was used to explore the relationship between variables. Hierarchical multiple regression was performed to assess the ability of measures including personality dimensions, spirituality, and coping strategy to predict satisfaction with clinical clerkship. Preliminary analyses were conducted to ensure no violation of the assumption of normality, linearity, multicollinearity and homoscedasticity.

## Results

A total of 293 participants included females (172) and males (121) comprising 61 and 39%, respectively. The mean age and mean hours of clerkship per week were 21.2 ± .79 and 17.8 ± 1.87, respectively. The mean and standard deviation of the variables, as well as their correlation with clinical clerkship satisfaction among intern nursing students are presented in Table 1.

**Table 1:** Mean and standard deviation scores for studied variables and their relationship with students’ satisfaction with clinical clerkship

Variables	Mean	SD	Correlation
Neuroticism	34.59	7.22	−.24***
Extraversion	40.88	6.37	.43***
Openness	38.39	3.72	.10*
Agreeable	41.13	5.73	.39***
Consciousness	43.73	6.23	.40***
Spirituality	51.17	6.79	53***
Problem-focused coping	55.37	9.60	.48***
Emotion-focused coping	58.61	8.48	.16**
Satisfaction with clinical experiences	155.52	35.42	1

*p < 0.05

**p < 0.01

***p < 0.001

Given the maximum score that could be obtained in SCCQ was 252, the mean score of 155 in SCCQ showed that participants were more satisfied than the average that could be obtained. Results of Pearson correlation indicated there were significant relationships between the independent variables (personality dimensions, spirituality, and coping strategies) and clinical clerkship satisfaction among intern nursing students (*p* < .05). While neuroticism (of personality dimensions) was negatively correlated with dependent variable (satisfaction with clinical clerkship), all other variables were found to be positively associated with clinical clerkship satisfaction. A hierarchical multiple regression ascertained how much variance in clinical clerkship satisfaction could be accounted for by personality dimensions, spirituality and coping strategies (Table 2).

**Table 2:** Results of multiple Hierarchical regression analysis for prediction of satisfaction with clinical clerkship in nursing students

	Variables	B	S.E.	β	T	*P* value	R	R^2^	∆R^2^
Model 1	Personality dimensions								
	Neuroticism	.45	.27	.09	1.70	.091	.53	.28^*^	–
	Extraversion	1.22	.31	.22	3.96^*^	.000			
	Openness	−.86	.45	−.09	−1.89	.059			
	Agreeable	.27	.39	.04	.69	.489			
	Consciousness	.43	.34	.08	1.27	.206			
Model 2	Spirituality	1.30	.27	.32	4.87^*^	.000	.59	.35^*^	.07^*^
Model 3	Coping strategies								
	Problem-focused	.97	.22	.26	4.44^*^	.000	.65	.42^*^	.07^*^
	Emotion-focused	.18	.22	.04	.82	.412			
	Constant	−45.64	28.28		−1.61	.108			

*p < 0.001

Personality dimensions were fed in the first step; spirituality was entered in the second step and coping strategies in the third step. Regarding clinical clerkship satisfaction, 28% of variances was accounted for in step 1 (*p* < .001). An additional 7% of the variance in clinical clerkship satisfaction was explained by the addition of the spirituality in step 2 (*p* < .001). Finally, an additional variance in clinical clerkship satisfaction accounted for by the addition of coping strategies in step 3 was 7% (*p* < .001). On the other hand, the full regression model explained 42% of the total variance in clinical clerkship satisfaction by personality dimensions, spirituality and coping strategies (*p* < .001). As shown, amongst the subscales of the personality questionnaire, only extraversion significantly predicted the dependent variable (β = 0.22, *p* < 0.001). In addition, the same significant predictive ability of spirituality (β = 0.32, *p* < 0.001) and problem-focused coping style for clinical clerkship satisfaction was found (β = 0.26, *p* < 0.001).

Two separate hierarchical regression analyses were conducted to determine how much variance in problem-focused and emotion-focused coping strategies could be explained by personality dimensions and spirituality. The results showed 21% of variance in problem-focused coping was explained by personality dimensions and spirituality (Table 3). The proportion of variance of marital satisfaction was accounted for by personality dimensions in the first step as 18% (*p* < .001) and by spirituality in the second step as 3% (*p* < .01). The beta coefficients revealed that openness (β = 0.14, *p* < 0.01), extraversion (β = 0.16, *p* < 0.01), and spirituality (β = 0.23, *p* < 0.001) could significantly predict the problem-focused coping style among intern nursing students. Hierarchical regression analyses for emotion-focused as a dependent variable showed that personality dimensions and spirituality were not able to significantly predict any portion of the variance changes. The full regression model explained 16% of the total variance in emotion-focused coping by personality dimensions and spirituality which was not significant (*p* > .05).

**Table 3:** Results of multiple Hierarchical regression analysis for prediction of problem-focused coping in nursing students

	Variables	B	S.E.	β	T	*P* value	R	R^2^	∆R^2^
Model 1	Personality dimensions								
	Neuroticism	.02	.08	.01	.25	.806	.43	.18^**^	–
	Extraversion	.25	.10	.16	2.58^*^	.010			
	Openness	.36	.14	.14	2.53^*^	.012			
	Agreeable	.05	.12	.03	.39	.698			
	Consciousness	.13	.11	.08	1.23	.218			
Model 2	Spirituality	.25	.08	.23	3.09^**^	.002	.46	.21^**^	.03^*^
	Constant	8.78	8.30		1.06	.291			

*p < 0.01

**p < 0.001

## Discussion

As noted in the result section, the mean score of the participants in the SCCQ was higher than the achievable average. It is possible that the proper use of coping styles, the adaptation of clinical environment characteristics with personality traits, and the spiritual view of caring patients have increased the readiness of students to enter the clinical clerkship. Although it is impossible to draw causal conclusions from a correlational study, the evidence suggests having the characteristics that prepare a student to deal with the problems of the clinical clerkship can reduce the “reality shock” and increase the clinical clerkship satisfaction [40].

All the variables presented in the study were positively correlated with the students’ clinical clerkship satisfaction despite the fact that neurosis (of personality dimensions) was negatively correlated. Findings are in concert with the previous studies [8, 11, 41].

According to the previous studies and the logical exception, all the correlations with students’ clinical clerkship satisfaction were sensibly assumed, except the positive correlation of emotion-focus coping which was presumed to be negative considering the previous literature [42, 43]. However, the use of emotion-focused coping strategies does not always have a negative consequence such as increasing stress. As an instance, under uncontrollable situations, this coping style can be helpful [25]. Due to the low experience of nursing students upon entering the clinical clerkship, they are likely to face many uncontrollable situations that require the use of emotion-focused coping strategy [8, 26]. The temporary use of emotion-focused coping in situations which are not controllable, such as experiencing a heart attack or waiting to undergo a surgery can be beneficial. Nonetheless, the case is different with long-run use of it as a special coping style [25]. As a result, a negative correlation between using emotion-focused coping style in students and satisfaction with their clinical clerkship cannot be taken for granted and the obtained results might be due to the situations in which the participants in the present study faced.

The results of hierarchical regression analysis showed (Hypothesis 1) personality dimensions which were fed in the first step for predicting clinical clerkship satisfaction in nursing students had a significant role. Among the five factors of personality, only extraversion predicted satisfaction with clinical clerkship of intern nursing students. Considering the definition of extraversion [13], students who were sociable, others’ lover and enjoyed talking to patients and also, those who had a tendency to acquire various experiences from their clinical practice, were more satisfied with their clinical clerkship [44, 45]. Nurses who are more extroverted are more likely to participate in teamwork and solve problems with the help of their colleagues. This in turn leads to increased clinical performance [46] and then satisfaction with the clinical course. However, as the length of the clinical clerkship increases, students become more skilled at communicating with patients, other nurses and their supervisors. As a result, they become more extroverted leading to their better performance [8].

The results of regression analysis showed that spirituality predicts satisfaction with clinical clerkship among intern nursing students (Hypothesis 1). In other words, considering the definition of spirituality, the students who found nursing as a meaningful and sacred career were more satisfied with their clinical clerkship. These findings are consistent with the previous studies [23, 47, 48]. The results of one study indicates that Iranian nurses consider helping patients an act of worship and believe that this contribution brings them spiritual rewards [22]. Evaluation of job values and job satisfaction among neophyte nurses [7], revealed “philanthropic” values play an important role in nurses’ job satisfaction. Additionally, those nurses who view their job as an opportunity to help others have more satisfaction with their job. It seems that among Iranian students of nursing, some spiritual values penetrate into job values and cause an increase in their satisfaction with clinical practice [48]. Although this finding was expected, it cannot be declared that spirituality is always associated with positive outcomes in caregivers [49, 50] and cannot necessarily predict their satisfaction [50].

Finally, the results showed problem-focused coping can predict satisfaction with clinical clerkship and emotion-focused coping have no role to play (Hypothesis 1). Such a finding is consistent with the available evidence [42, 51]. In line with the definition of problem-focused coping, the students who try to encounter reality shock and the factors producing stress in clinical settings and find a way around it are more satisfied with their clinical clerkship compared with those who are motivated by excitation of stressful events. Students who are able to deal with the problems of the clinical clerkship and use designated problem-solving strategies outperform those who engage in dependent behaviors such as indisputable agreement with the decision made by others. They use knowledge and experience to identify or manage patient care problems, but do not expect the supervisor to solve their clinical problems. Therefore, they have better evaluation about their clinical performance [52]. However, it seems as the length of the clinical clerkship increases, students gain more professional competencies and use more problem-oriented coping [8, 29].

The hierarchical regression analysis showed personality dimensions and spirituality can significantly predict problem-focused coping of intern nursing students (Hypothesis 2). Among the personality dimensions, extraversion and openness could predict problem-focused coping. The findings are consistent with the previous studies [30, 53]. Extraversion, a general tendency to be assertive, is defined as being active and engaging in social interactions. These individuals tend to be cheerful and, therefore, it seems logical to have good relations with others [13] and seek social support as a subpart of problem-focused coping style [25]. Openness (to clerkship experiences) is also defined as the tendency to ponder novel ideas, unconventional values, and divergent thinking [13]. Therefore, it can be assumed that individuals with high scores in openness are flexible, creative, and capable of exploiting a number of more efficient coping strategies to deal with distressing situations [25]. In general, extraversion increases the openness to new experiences in clinical settings by improving the students’ communications with patients, other students and supervisors, thereby allowing students to cope with clinical problems by considering new experiences in a problem-focused manner [53].

It was also found that spirituality predicted problem-focused coping. In other words, students who were able to find meaning and sanctity in their clinical clerkship were more likely to use problem-oriented coping. A few studies have been carried out on the relationship between spirituality and general coping strategies, most of which refer to the role of spirituality as a way to cope with stress as “spiritual coping” [54, 55]. Spirituality can help gather or focus resources on problem solving. Hence, spiritual coping is expected to have a closer relationship with the problem- focused coping [56].

In coping with stress, three roles are considered for spirituality: 1) providing a meaning for life, 2) helping people to have a sense of control in various situations, and 3) improving self-esteem in coping with stressful situations [57]. The role of spirituality in predicting problem-focused coping style for nursing students can be justified by the fact that spirituality gives students a sense of control and confidence in working with real patients so that they can seek to find solutions to problems. In other words, spirituality includes a wide range of personal, spiritual and existential beliefs that may be utilized in dealing with stress, leading a person to use problem-oriented coping; therefore, spirituality can improve life satisfaction by increasing the use of problem-focused coping styles. The problem-focused coping strategies can further play a mediating role in the relationship between spirituality and life satisfaction [58].

Finally, the results of regression analyses showed personality dimensions and spirituality were not able to significantly predict emotion-focused coping in nursing students (Hypothesis 3). The findings are inconsistent with the previous studies [38, 53]. This finding can be attributed to students’ low use of emotion-oriented coping strategies. Evidence suggests nursing students tend to use more problem-oriented coping strategies in clinical situations than emotion-focused ones [29, 51]. Consequently, regardless of personality traits or spirituality, students may not have a tendency to use emotion-oriented coping strategies. On the other hand, the duration of the clinical clerkship in this study was not controlled while the students with more clinical experience may be more likely to use problem-oriented coping situations than the emotion-focused ones [8].

## Conclusion and recommendation

Based on the findings, it can be concluded that spirituality is an important factor in predicting clinical clerkship satisfaction and problem-focused coping among nursing students. Therefore, paying attention to spiritual needs of nursing students can be effective both in better coping with the stressful situations of clinical setting and in enhancing their satisfaction with clinical clerkships. Also, given the role of problem-focused coping in predicting satisfaction with clinical clerkship, helping students to resolve their clinical problems rationally and enhancing their problem solving skills in dealing with clinical stresses can be associated with their clinical clerkship satisfaction. Considering the role of extraversion in predicting the satisfaction with clinical clerkship and problem-focused coping in nursing students, it can be concluded that the training of social behaviors, communication skills and proper interaction with patients and hospital staff (extraversion characteristics) can also help nursing students to effectively cope with stressful work situations and also increase their clinical clerkship satisfaction.

### Limitations

Considering the correlational design of the study, it is not possible to draw any causal conclusions from the findings. In order to better generalize the findings of this study, especially regarding limited studies on the role of spirituality in clinical setting and the spiritual needs of nursing students, further similar studies should be conducted. Given spirituality [59] and coping strategies [60] are to some extent dependent on ethnicity, the generalizations of the research findings to other ethnic groups should be made with caution and it is suggested that similar studies are conducted covering other ethnic groups. This cross-sectional study was conducted to assess the factors predicting clinical clerkship satisfaction. To this end, further studies can employ longitudinal methods to assess such factors.

## Data Availability

Data and materials are confidential but they will be available upon reasonable request from the corresponding author.

## Abbreviations

NEO_FFI: NEO five factor inventory
WoCQ: ways of coping questionnaire
